# DNA-Damage Foci to Detect and Characterize DNA Repair Alterations in Children Treated for Pediatric Malignancies

**DOI:** 10.1371/journal.pone.0091319

**Published:** 2014-03-17

**Authors:** Nadine Schuler, Jan Palm, Mareike Kaiser, Dominik Betten, Rhoikos Furtwängler, Christian Rübe, Norbert Graf, Claudia E. Rübe

**Affiliations:** 1 Department of Radiation Oncology, Saarland University, Homburg/Saar, Germany; 2 Department of Pediatric Hematology and Oncology, Saarland University, Homburg/Saar, Germany; Dresden University of Technology, Germany

## Abstract

**Purpose:**

In children diagnosed with cancer, we evaluated the DNA damage foci approach to identify patients with double-strand break (DSB) repair deficiencies, who may overreact to DNA-damaging radio- and chemotherapy. In one patient with Fanconi anemia (FA) suffering relapsing squamous cell carcinomas of the oral cavity we also characterized the repair defect in biopsies of skin, mucosa and tumor.

**Methods and Materials:**

In children with histologically confirmed tumors or leukemias and healthy control-children DSB repair was investigated by counting γH2AX-, 53BP1- and pATM-foci in blood lymphocytes at defined time points after *ex-vivo* irradiation. This DSB repair capacity was correlated with treatment-related normal-tissue responses. For the FA patient the defective repair was also characterized in tissue biopsies by analyzing DNA damage response proteins by light and electron microscopy.

**Results:**

Between tumor-children and healthy control-children we observed significant differences in mean DSB repair capacity, suggesting that childhood cancer is based on genetic alterations affecting DNA repair. Only 1 out of 4 patients with grade-4 normal-tissue toxicities revealed an impaired DSB repair capacity. The defective DNA repair in FA patient was verified in irradiated blood lymphocytes as well as in non-irradiated mucosa and skin biopsies leading to an excessive accumulation of heterochromatin-associated DSBs in rapidly cycling cells.

**Conclusions:**

Analyzing human tissues we show that DSB repair alterations predispose to cancer formation at younger ages and affect the susceptibility to normal-tissue toxicities. DNA damage foci analysis of blood and tissue samples allows one to detect and characterize DSB repair deficiencies and enables identification of patients at risk for high-grade toxicities. However, not all treatment-associated normal-tissue toxicities can be explained by DSB repair deficiencies.

## Introduction

Over the last decades, increasingly complex multimodality treatment protocols have led to tremendous improvements in the survival of children diagnosed with cancer. However, all DNA-damaging cancer therapies are associated with early and late adverse effects, also known as normal-tissue toxicities. DNA double-strand breaks (DSBs), the most deleterious DNA lesions, are produced to a great extent when cells are exposed to DNA-damaging agents, such as ionizing radiation and certain chemotherapeutics. Normal-tissue responses show considerable variability among patients, whereas treatment-associated complications are not only related to the specific therapy used but may particularly be determined by the patient's individual genetic predisposition. Most convincing evidence suggests that genetic alterations in proteins participating in the DNA damage response determine the individual risk of developing severe treatment-related side effects [1], [2].

DSBs are repaired by two major pathways: non-homologous end joining (NHEJ) or homologous recombination (HR). NHEJ repairs DSBs in all cell-cycle phases and represents the major pathway in G1, while HR functions in S/G2. NHEJ involves the binding of the heterodimeric Ku protein to double-stranded DNA ends, recruitment of the DNA-dependent protein kinase catalytic subunit (DNA-PKcs) to generate the DNA-PK holoenzyme and DNA-PK kinase activation. The assembled DNA-PK complex on the DNA end then helps to recruit a complex involving DNA ligase IV and XRCC4, which effects the rejoining step [3]. HR is a more complex process involving 5′–3′ end resection to generate a 3′ single-stranded (ss) DNA overhang [4]. The ssDNA is rapidly bound by RPA, which is subsequently displaced by RAD51 in a process that involves BRCA2. Invasion of a homologous sequence to generate a Holliday junction and heteroduplex DNA then follows. Subsequent steps involve branch migration, fill-in of the ssDNA regions and Holliday junction resolution [5].

Several proteins involved in DNA damage signaling produce discrete foci in response to ionizing radiation, and the visualization of DNA-damage foci such as γH2AX (phosporylated histone H2AX) or 53BP1 (53 Binding Protein 1) by fluorescence microscopy has been used to quantify radiation-induced DSBs and elucidate DNA repair pathways and foci kinetics have been used as a tool to assess individual radiosensitivity [6],[7],[8],[9]. However, core members of the NHEJ pathway (including Ku70-Ku80 heterodimer) do not visibly accumulate in foci because they are only required at low copy number. Recently, we established a gold-labeling technique for identification and localization of different DNA repair components within the cell nuclei of tissue samples using transmission electron microscopy (TEM) [10]. The high resolution of TEM permits visualization of the intracellular distribution of repair proteins at the single-molecule level within subnuclear compartments. Intriguingly, TEM visualization of phosphorylated Ku70 (pKu70), which binds directly to broken DNA ends in preparation for rejoining, permitted reliable detection of unrepaired DSBs in euchromatic and heterochromatic domains [10], [11].

Rare hereditary diseases with defects in DNA repair such as Ataxia telangiectasia and Fanconi anemia have in common an increased risk of cancer and a premature aging phenotype [12]. Ataxia telangiectasia (AT) is a neurodegenerative autosomal recessive inherited disease caused by a defect in the ataxia–telangiectasia mutated (ATM) gene [13]. The ATM kinase integrates the cellular response to DSBs by phosphorylating key proteins involved in cell-cycle regulation and DNA repair, and thus plays a central role in the maintenance of genomic integrity [14]. Recent work by Jeggo and co-workers has demonstrated that the chromatin environment at a DSB significantly impacts upon DSB repair and that ATM sigalling enhances relaxation of heterochromatin in the DSB vicinity to facilitate repair [15], [16], [17]. Whereas carriers of monoallelic ATM mutations (ATM^+/−^ heterozygote) have slightly elevated cancer risks and increased radiosensitivity, the complete lack of ATM function results in the clinical syndrome AT (ATM^−/−^ homozygote), characterized by progressive neuromotor dysfunction, immunodeficiency, genomic instability, predisposition to cancer, and profound hypersensitivity to ionizing radiation [18].

Fanconi anemia (FA) is an X-linked or autosomal recessive inherited disease becoming manifest in the first decade of childhood with infantile aplastic anemia [19]. In the subsequent course of the disease, patients develop tumors such as lymphomas, esophageal carcinomas, squamous cell carcinomas of the head and neck and acute myelogenous leukaemia. The skin shows a poikilodermatic appearance with hypo- and hyperpigmentation and telangiectasia. Mutations in several genes can give rise to FA and patients are classified in complementation groups, depending on which gene is mutated. Together, FA proteins form a DNA damage activated signaling pathway, the so called FA pathway, which has recently been shown to promote replication-dependent DNA interstrand crosslink repair [20], [21].

DNA repair disorders such as AT or FA are rare, and malignancies arising in the context of cancer predisposition syndromes account for only 5–10% of human tumors [22]. However, minor DSB repair deficiencies based on subtle heterogenous mutations in DNA damage response genes are expected to be more common in the human population. In a clinical trial with children suffering pediatric malignancies, we evaluated the potential of the DNA-damage foci approach to identify patients with DSB repair deficiencies who may overreact to DNA-damaging cancer therapy. In previous experimental studies, we could show that γH2AX-foci analysis of blood lymphocytes provides precise information about the genetically-defined DSB repair capacity, shown to be valid for different and complex organs in a given individual [23]. Moreover, in previous clinical trials the γH2AX-analysis allowed us to verify substantial DSB repair defects in patients displaying severe side effects after radiotherapy, sustaining the close relationship between DSB repair deficiency and pronounced clinical radiosensitivity [24].

In the present study, we used different DNA damage marker to evaluate the foci approach to identify patients with DSB repair deficiencies as a screening tool in predictive testing for normal-tissue toxicities. At first, we analyzed blood lymphocytes of children with AT (ATM^−/−^ homozygote) and their heterozygous parents (ATM^+/−^ heterozygote) to evaluate the feasibility of the different DNA-damage marker to verify not only pronounced but also subtle, genetically-defined DSB repair deficiencies. Subsequently, we used this approach to investigate the DSB repair capacity of 41 children with different pediatric malignancies and monitored their treatment-associated normal-tissue responses. Moreover, in one patient with FA undergoing surgical procedures for head and neck tumor, the functional defect in the FA pathway could also be validated in tissue specimens, with an excess of unrepaired DSBs in the proliferating zone of the mucosa and skin.

## Materials and Methods

### Probands and patients

Twelve different families with 16 ATM^−/−^ homozygote children/adolescents and 16 ATM^+/−^ heterozygote parents were analyzed compared to 5 normal ATM^+/+^ individuals (n = 37). Some of the underlying ATM gene mutations have been identified previously [24]. Children with histologically confirmed tumors or leukemias, who received chemo- and/or radiotherapy in our departments between May 2009 and July 2012, were included in this study (n = 41, listed in table 1). Exclusion criteria were previous radio-/chemotherapy within the last 3 months. Treatment-associated normal-tissue toxicities were documented according to the “Registry for the Evaluation of Late Side Effects after Radiation in Childhood and Adolescence” (RiSK) protocol [25].

**Table 1:** Characteristics of the children with tumors.

	ID	age at diagnosis (y)	sex	cancer histology	radiotherapy	DNA-damaging chemotherapy	acute side effects (≥ grade 3)	late side effects (≥ grade 3)	findings supporting genetic predisposition
1	BRBY	2	F	Astrocytoma	-	-	-	-	none
2	BRNO	3	M	Non-Hodgkin's lymphoma	-	DNR, CPM, DOX	mucositis, neutropene fever	-	none
3	BNLC	7	M	Osteosarcoma	-	DOX, CDDP	-	-	none
4	BUEI	7	F	Nephroblastoma	-	DOX, DACT-D, IFO, CBDCA, VP-16, CPM, TOPO, TMZ	-	-	none
5	BRRN	7	M	Non-Hodgkin's lymphoma	-	-	mucositis, neutropene fever	-	none
6	CAFO	2	M	Acute lymphocytic leukemia	-	DNR, CPM	-	-	none
7	DRLE	12	F	Glioblastoma	site of primary tumor (59.4 Gy)	TMZ	-	-	secondary leukemia, brother died from glioblastoma
8	DRMN	5	M	Acute lymphocytic leukemia	-	DOX, CPM	-	-	none
9	EKSI	6	M	Neuroblastoma	-	CPM, TOPO, TMZ, DTIC, IFO, DOX, VP-16, CBDCA, CDDP	mucositis, neutropene fever	Nn. optici atrophy, high-frequency hearing loss	none
**10**	**GRJN**	**5**	**M**	**Medulloblastoma**	**craniospinal axis (35.2 Gy), fossa posterior (55 Gy)**	**CBDCA, VP-16, TMZ**	**encephalopathy after intrathecal MTX**	**-**	**none**
11	GSLA	14	F	Osteosarcoma	-	DOX, CDDP	-	osteoporosis, pathological fracture	none
12	HNLT	2	M	Nephroblastoma	renal bed (15Gy), whole-lung irradiation (15 Gy)	DOX, DACT-D, VP-16, CBDCA	-	-	none
13	HZFC	22	M	Acute lymphocytic leukemia	-	IDA, CPM, DNR	-	-	trisomy 21 (hyposomia, syndactyly, thyroid hypofunction)
**14**	**HFLA**	**15**	**F**	**Acute lymphocytic leukemia**	**-**	**DOX, CPM, DNR**	**toxic hepatopathy, neuropathy, osteonecrosis, sepsis**	**-**	**none**
15	HNLA	14	F	Astrocytoma	-	-	-	-	none
16	HRTS	1	M	Retinoblastoma	orbital region (50 Gy)	-	-	-	brother suffered from osteosarcoma
17	KIDA	7	F	Osteosarcoma	-	CDDP, DOX	-	muscular contractions, telipes equinus	none
18	KSLS	4	M	Chronic myeloid leukemia	total-body irradiation (12 Gy)	DNR, CPM	mucositis, enterocolitis, prolonged aplasia	GvHD (skin, liver), cataract	Philadelphia chromosome
19	KNML	17	M	Acute lymphocytic leukemia	-	DNR, CPM	toxic hepatopathy	-	none
20	KSVA	15	F	Acute lymphocytic leukemia	preventive cranial irradiation (12 Gy)	IDA, VP-16, CPM, DNR, IFO	mucositis, transitoric ischaemic attack	-	Philadelphia chromosome
21	KNCA	3	F	Acute lymphocytic leukemia	-	DNR, CPM	-	-	none
22	KLVA	11	F	Acute lymphocytic leukemia	total body irradiation (12 Gy)	DNR, CPM	prolonged aplasia	-	Philadelphia chromosome
23	KTMZ	12	M	Germ cell tumor	-	CDDP, VP-16, IFO	-	-	none
24	LEDN	4	M	Rhabdoid tumor	renal bed (19.8 Gy), whole-lung irradiation (12 Gy)	CBDCA, DOX, IFO, VP-16, CPM, DACT-D	mucositis, enterocolitis	-	none
25	MRAN	13	M	Hodgkin's disease	-	CPM	-	-	none
26	MACR	5	M	Anaplastic ependymoma	craniospinal axis (39.6 Gy), fossa posterior (68Gy), cerebral lesions (30 Gy)	TMZ, VP-16, CPM, CBDCA	-	-	none
27	MKTS	14	M	Hodgkin's disease	-	VP-16, ADR	-	-	none
28	MRNO	5	M	Rhabdomyosarcoma	site of primary tumor (41.4 Gy)	IFO, DOX, VP-16, DACT-D	-	-	none
**29**	**MRME**	**14**	**F**	**Acute lymphocytic leukemia**	**total-body irradiation (12 Gy)**	**DNR, CPM, IFO**	**neutropene sepsis, cardiomyopathy**	**-**	**none**
30	NZBT	6	M	Acute lymphocytic leukemia	total-body irradiation (12 Gy)	IDA, DNR, CPM	mucositis, enterocolitis, neutropene fever	GvHD (intestine)	trisomy 11 and 22 (dysmorphic features, skeletal abnormalities, hypoplastic kidney)
31	PUJN	5	M	Glioma of N. opticus	-	-	-	-	neurofibromatosis type II
32	PLIE	1	F	Ewing's sarcoma	-	DACT-D, IFO, CPM, DOX, VP-16	-	-	none
33	RSML	17	M	Osteosarcoma	-	IFO, VP-16, CDDP, DOX	Lyell syndrome after MTX, enterocolitis, hepatopathy	-	none
**34**	**SRAA**	**8**	**F**	**Acute lymphocytic leukemia**	**total-body irradiation (12 Gy), lesions with leukemic infiltrations (20–36 Gy)**	**CPM, VP-16, DOX, IDA, IFO, CBDCA**	**mucositis, neutropene sepsis**	**GvHD (skin, liver), neuropathy**	**none**
35	HYSD	1	M	Supratentorial PNET	craniospinal axis (24 Gy), right frontal lobe (54.6 Gy)	CBDCA, VP-16	chemotherapy-induced cerebrospinal fluid blockage with increased intracranial pressure	-	none
36	SKAE	5	F	Nephroblastoma	-	DOX, DACT-D, VP-16, CBDCA	-	-	none
37	SRMA	4	F	Rhabdomyosarcoma	primary and metastatic lesions (45 Gy)	IFO, DACT-D, CBDCA, VP-16, TRO, IDA, EPI	mucositis, neutropene fever	-	none
38	**SBNE**	**13**	**F**	**Relapsing squamous cell cancers of the oral cavity**	**-**	**-**	**-**	**-**	**Fanconi anemia (FANC-L) with microsomia, microcephaly, skeletal abnormalities, dystopy of the kidney**
39	SSJA	8	F	Acute lymphocytic leukemia	preventive cranial irradiation (12 Gy)	IFO, DNR, IDA, CPM, VP-16	prolonged aplasia, enterocolitis, hemorrhagic cystitis	cataract	Philadelphia chromosome
40	WRBN	15	M	Pheochromocytoma	-	-	-	-	none
41	YZMD	10	M	Acute lymphocytic leukemia	-	DOX, CPM, DNR	-	-	none

Abbreviations: TMZ: Temodal, CCNU: Lomustin, VP-16: Etoposid, MTX: Methotrexat, IFO: Ifosfamid, DOX: Doxorubicin, CPM: Cyclophosphamid, CDDP: Cisplatin, DACT: Actinomycin-D, TOPO: Topotecan, DNR: Daunorubicin, DTIC: Dacarbacin, CBDCA: Carboplatin, IDA: Idarubicin, ADR: Adriamycin, TRO: Trofosfamid, EPI: Epirubicin.

### Ethics statement

Protocol procedures were approved by the local ethics committee (“Ethikkommission der Ärztekammer des Saarlandes”), and all children as well as their parents provided written informed consent.

### DNA-damage foci analysis in blood lymphocytes

Peripheral blood was collected in sodium heparin-containing vacutainers and lymphocytes were isolated immediately by density gradient centrifugation (Percoll, PAA). After DMSO (Carl Roth GmbH, Karlsruhe, Germany) addition lymphocytes were placed in a freezer for slow freezing to −80°C. After two days at −80°C, samples were transferred to liquid nitrogen vapor phase for long-term storage. After 6–12 months of cryopreservation, defrosted blood lymphocytes were irradiated with 1Gy or 2Gy (X-ray: 90 kV, 19 mA; dose-rate: 1 Gy/min), respectively. At 0.25 h, 8 h or 24 h post-irradiation lymphocytes were spotted onto coverslips. Samples were fixed in methanol, permeabilized in acetone and incubated with anti-γH2AX (Upstate, Charlottesville, VA, USA) anti-53BP1 (Bethyl, Montgomery, TX, USA) or anti-pATM (Abcam, Cambridge, UK) followed by Alexa-Fluor-488 conjugated goat-anti-mouse secondary antibody (Invitrogen, Karlsruhe, Germany). Afterwards, samples were mounted in VECTAshield with 4′,6-diamidino-2-phenylindole (DAPI) (VectorLaboratories, Burlingame, CA, USA). For double-labeling, samples were incubated with anti-γH2AX, anti-53BP1 or anti-pATM antibodies followed by Alexa-Fluor-488 and Alexa-Fluor-568 conjugated secondary antibodies (Invitrogen, Karlsruhe, Germany). Using E600-epifluorescent microscope (Nikon, Düsseldorf, Germany) DNA-damage foci per cell were counted by eye until at least 40 cells or 40 foci were scored for each data point.

### Immunohistochemical analysis of tissue specimens

Immunohistochemical staining for RAD51, and 53BP1 was performed using the DakoEnVisionTM kit (Dako, Glostrup, Denmark). After dewaxing in xylene and rehydration in graded alcohols, tissue sections were boiled in citrate buffer. Sections were incubated with primary antibody. And staining was completed by incubation with 3,3-diaminobenzidine (DAB) and substrate chromogen, which results in brown-colored precipitate at the antigen site. Finally, sections were counterstained with haematoxylin and mounted in Aqueous Mounting Medium (Dako, Glostrup, Denmark).

### Immunofluorescence analysis of tissue specimens

Formalin-fixed tissues were embedded in paraffin and sectioned at a thickness of 4 μm. After dewaxing in xylene and rehydration in decreasing concentrations of alcohol, sections were boiled in citrate buffer and pre-incubated with Roti-Immunoblock (Carl Roth, Karlruhe, Germany). Sections were then incubated with the primary antibody (anti-53BP1, Bethyl Laboratories, Montgomery, TX, USA), followed by Alexa Fluor-488 secondary antibody (Invitrogen, Karlsruhe, Germany). Finally, sections were mounted in VECTAshield with 4′,6-diamidino-2-phenylindole (DAPI; Vector Laboratories, Burlingame, CA, USA). For quantitative analysis, 53BP1-foci were counted visually under a Nikon E600 epifluorescent microscope (Nikon, Düsseldorf, Germany) using objective magnification of ×60. Counting of 53BP1-foci was performed until at least 40 foci or 40 cells were registered for each data point.

### TEM analysis of tissue specimens

Tissue samples were diced into small cubes of 2 mm^3^ and fixed overnight with paraformaldehyde and glutaraldehyde. Fixed tissue samples were dehydrated in increasing concentrations of alcohol and infiltrated with LR Gold resin (EMS, Hatfield, PA). Finally, samples were embedded in fresh resin with benzil (EMS) and polymerizated with ultraviolet light illumination. Ultrathin sections were cut on an Ultracut UCT Leica with diamond knives (Diatome; Biel, Switzerland), picked up with pioloform-coated nickel grids, and processed for immuno-labeling. To block unspecific staining, sections were floated on glycine followed by blocking solution (EMS). Afterwards, sections were incubated with the primary antibody (anti-53BP1, Bethyl Laboratories; anti-pKu70 (pSer6), AbcamInc, Cambridge, MA, USA). After rinsing, secondary antibody conjugated with 6-nm or 10-nm goldparticles (EMS) was applied to the grids. Subsequently, sections were rinsed and post-fixed with glutaraldehyde. Immunogold-labeling omitting the primary antibody was used as control. All sections were stained with uranyl acetate and examined using a TecnaiBiotwin transmission electron microscope (FEI Company, Eindhoven, The Netherlands).

### Statistical analysis

All experiments have been performed at least 2 times for each individual. To evaluate potential differences in DSB repair capacity, statistical comparisons were performed at 8 h and 24 h postirradiation by Mann-Whitney test, respectively, using OriginPro software. Criterion for statistical significance was p≤0.05.

## Results

**Figure 1:**
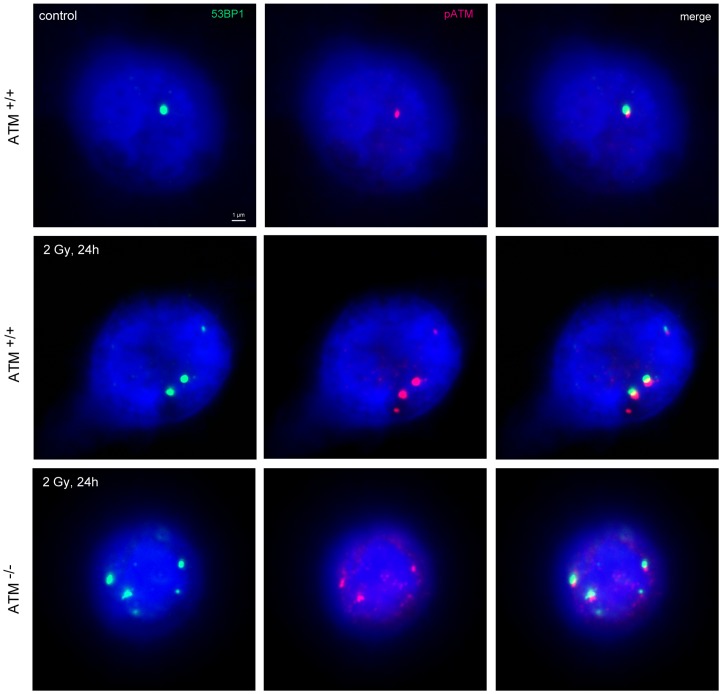
DNA damage foci in blood lymphocytes. Immunofluorescence double staining of 53BP1 (green) and pATM (red) in blood lymphocytes of healthy individuals (ATM^+/+^) and ATM^−/−^ homozygotes, analysed 24 h after irradiation with 2Gy compared to un-irradiated control. Deoxyribonucleic acid was counterstained with 4′,6-diamidino-2-phenylindole (blue), and images were merged to determine co-localization (yellow). Original magnification, x600.

The DSB repair capacity of ATM^−/−^ homozygote and ATM^+/−^ heterozygote probands was evaluated to test sensitivity and reliability of the DNA-damage foci approach in the clinical setting to identify DSB repair deficiencies. Blood lymphocytes were irradiated with either 1Gy to determine the maximal induction at 15 min (0.25 h) or with 2Gy to capture the foci loss within 8 h and 24 h after radiation exposure. At these defined time-points samples were fixed, stained for γH2AX, 53BP1 and pATM, and examined by fluorescence microscopy for enumeration of foci per cell. Our results showed that γH2AX-, 53BP1- and pATM-foci co-localize even in the ATM^−/−^ homozygote individuals, underscoring that counting these DNA damage foci can be used to analyze DSB repair (Fig. 1).

**Figure 2:**
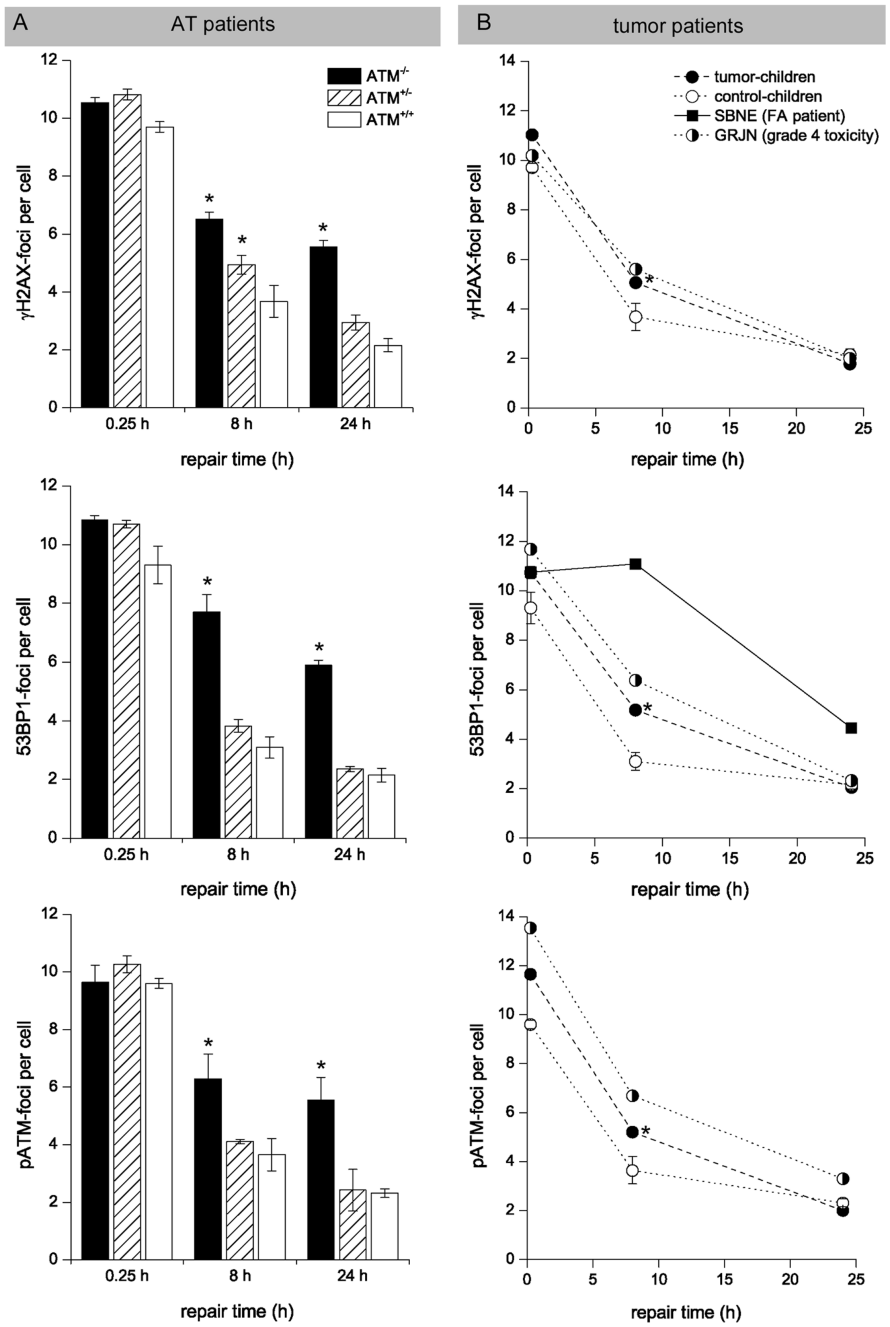
Foci kinetics measured in blood lymphocytes. Double-strand break repair kinetics of ATM^−/−^ homozygotes, AMT^+/−^ heterozygotes (A), and tumor-children (B) compared to healthy control-children (ATM^+/+^) evaluated by counting γH2AX-, 53BP1- and pATM-foci in blood lymphocytes at defined time-points after irradiation. GRJN developing grade-4 toxicities during multimodality treatment for medulloblastoma and SBNE with FA suffering relapsing squamous cell carcinomas of the oral cavity revealed impaired DSB repair capacities. * significant difference to healthy control-children (p≤0.05).

The time-course of foci loss in blood lymphocytes of ATM^−/−^ homozygote (n = 16), ATM^+/−^ heterozygote (n = 16), and normal individuals (ATM^+/+^, n = 5) after radiation exposure is shown in figure 2A. Lymphocytes from healthy individuals (ATM^+/+^) exhibited a rapid decrease in foci number within first hours postirradiation, and only low levels of damage were observed at 8 h (3.0–3.7 foci/cell) and 24 h postirradiation (2.1–2.3 foci/cell), with no significant differences between the various DNA damage marker. In contrast, ATM-deficient lymphocytes of ATM^−/−^ homozygotes showed a clearly slower decline with considerably increased foci numbers at 8 h (6.3–7.7 foci/cell) and 24 h (5.6–5.9 foci/cell) postirradiation, with more than 3 excess foci/cell compared with controls. Tests of significance for differences in mean values showed that all values obtained with the different markers for 8 h and 24 h samples from ATM^−/−^ homozygote probands were significantly different from corresponding values for normal individuals (p≤0.05).

The time-course for lymphocytes from ATM^+/−^ heterozygotes showed a different pattern of foci disappearance dependent on the DNA-damage marker used. Significantly, ATM^+/−^ heterozygous individuals showed slightly elevated levels of remaining γH2AX-foci at 8 h (3.83±0.22 foci/cell), but not at 24 h (2.36±0.09 foci/cell) post-irradiation. For the DNA-damage marker 53BP1 and pATM we observed similar values at 8 h and 24 h for the ATM^+/−^ heterozygous individuals compared to the healthy controls (ATM^+/+^). Collectively, these data indicate that analysing the kinetics for γH2AX-foci loss can be used to assess the DSB repair capacity in blood samples of individuals; however using 53BP1 or pATM as DNA-damage marker slight impairments in DSB repair caused by heterogeneous mutations were not detected reliably.

Subsequently, we analyzed the DSB repair of children with different pediatric malignancies (n = 41) in comparison to healthy control-children (n = 5). Childhood leukemia was included in this study to test whether malignant or immature leukocytes in peripheral blood may falsify results (percentage of blast cells was documented at blood withdrawal). In the majority of cases, blood samples were obtained at diagnosis, thus before any DNA-damaging chemo-/radiotherapy was applied. The characteristics of the 41 patients eligible for this study are summarized in table 1. The mean age of the tumor-children was 8.3 years. Of the 41 patients, 17 were female and 24 male. Most patients suffered from leukemias, followed by soft-tissue and bone sarcomas. 19 patients suffered severe normal tissues responses, therefrom 4 patients with grade 4 toxicities (GRJN; HFLA; MRME; SRAA; marked in Tab. 1)

According to the experimental protocol described above, blood lymphocytes of tumor-children were irradiated with 1Gy or 2Gy, respectively, and DSB repair kinetics were evaluated by DNA-damage foci analysis at 0.25 h, 8 h and 24 h post-irradiation (Fig. 2B). Strikingly, the average values of γH2AX-foci numbers for the tumor-children were significantly higher at 8 h post-irradiation (5.06±0.09 foci/cell) compared with values obtained for healthy controls (3.68±0.55 foci/cell; p≤0.05). A similar trend was observed analyzing 53BP1- and pATM-foci, suggesting a slightly impaired DSB repair capacity in children with malignancies. However, only patient (GRJN, marked in table 1) revealed clearly elevated foci levels for γH2AX and 53BP1 at 8 h and for pATM at 8 h and 24 h post-irradiation, with ∼1–3 excess foci per cell compared with average values of healthy controls, suggesting a slight DSB repair impairment.

**Figure 3:**
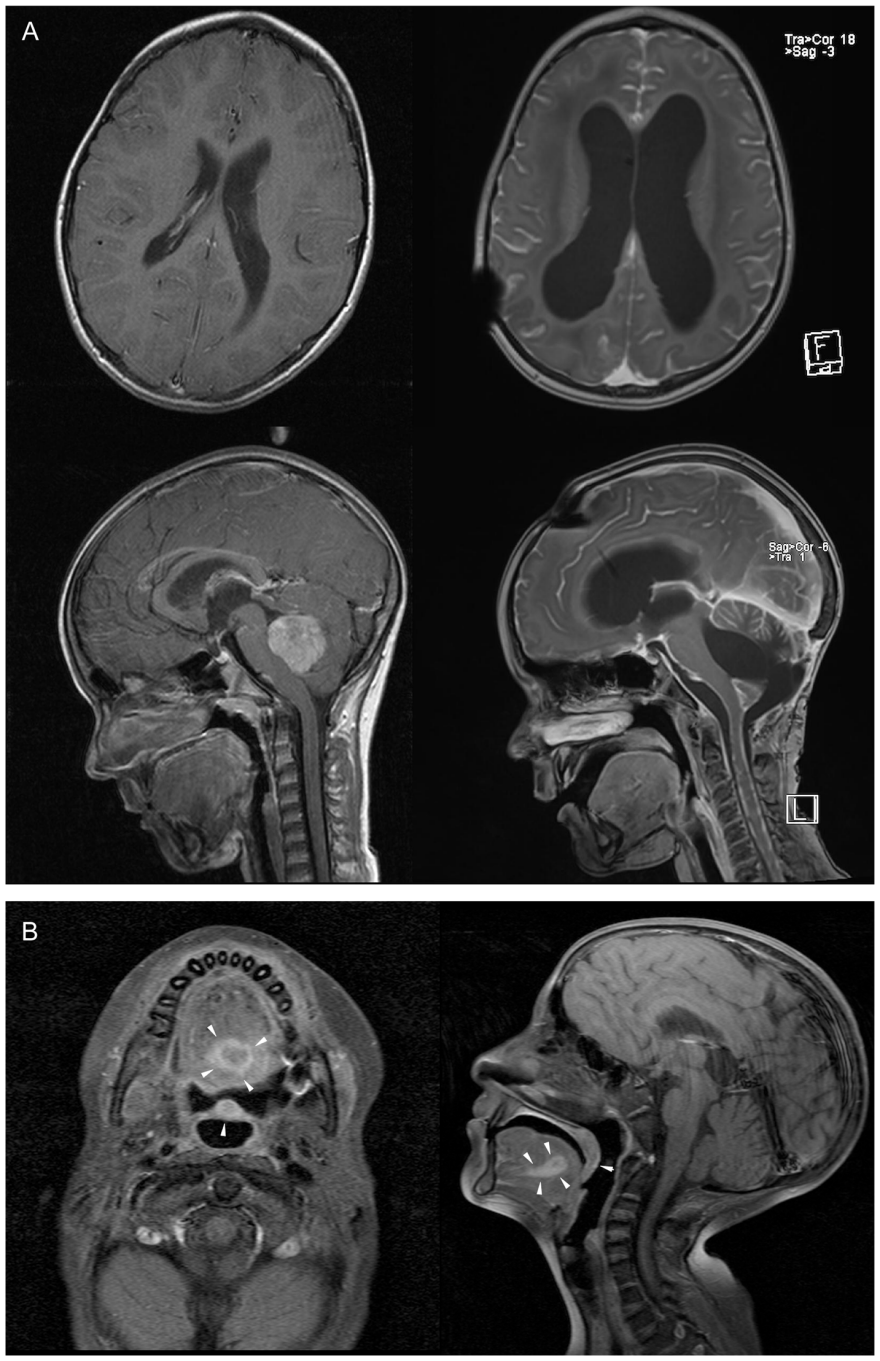
MR imaging documenting severe normal tissue toxicity or synchronous tumors in patients. (A) Cranial magnetic resonance imaging of the patient GRJN before (left panel) and after tumour resection and adjuvant radio-/chemotherapy (right panel). T1-weighted images acquired in sagittal and axial plane shows cerebral atrophy with enlargement of the ventricles after multifactorial leukencephalopathy. (B) Cranial magnetic resonance imaging of the patient SBNE before surgical tumor resection. T1-weighted images acquired in sagittal and axial plane reveal the squamous cell carcinoma (SCC) of the oral cavity (tumors marked by arrows).

The 5-year old GRJN was diagnosed with medulloblastoma in the cerebellar vermis and fourth ventricle obstructing the cerebrospinal fluid flow. After subtotal tumor resection and ventriculoperitoneal shunt placement the entire craniospinal axis was irradiated with 35.2 Gy, followed by a boost of 19.8 Gy to the primary tumor site in the fossa posterior. Adjuvant multidrug chemotherapy including carboplatinum and VP-16 revealed only partial response. After intrathecal methotrexate application GRJN developed a necrotizing leukoencephalopathy with somnolence, seizures, spasticity, and paresis treated by high-dose steroids and anticonvulsants. Cranial magnetic resonance imaging (MRI) revealed demyelinating lesions in periventricular regions with diffuse areas of white matter necrosis, enlargement of the ventricles, and cerebral atrophy with an increase in the sulcal width (Fig. 3A). Suffering therapy-refractory disease progression with tumor metastasis in the spinal subarachnoid space GRJN died 8 months after diagnosis. Collectively, the impaired DSB repair capacity of GRJN leading to an increased sensitivity to DNA-damaging therapy appears to be a contributing factor to the multifactoriel etiology of leukencephalopathy. However, none of the other children (HFLA, MRME and SRAA) suffering grade-4 toxicities during multimodality treatment revealed an impaired DSB repair capacity. Taken together, these findings suggest that an impaired DSB repair capacity is associated with an increased risk for high-grade toxicities, but not all severe normal-tissue responses can be explained by deficient DSB repair capacities.

In this clinical study we also analyzed blood lymphocytes of the FA patient SBNE suffering relapsing squamous cell carcinomas of the oral cavity to evaluate her DSB repair capacity to explore radio- and chemotherapeutic options for tumor eradication. The defective DNA repair mechanisms in FA patients make normal tissue more susceptible to short-term and delayed tissue effects of chemo- and radiotherapy. This increased susceptibility can present a problem in determining and delivering a cancericidal doses without causing significant damage to normal tissues. Due to congenital anomalies (growth retardation, skeletal deformities of the forearm, microcephaly, microphthalmia, dystopy of the kidney, endocrine abnormalities) SBNE was diagnosed with FA at the early infancy (FANC-L, an extremely rare FA subtype). During the first decade of life SBNE developed progressive bone marrow failure, treated successfully by hematopoietic stem cell transplantation with a histocompatible sibling donor. Repeated bone marrow examinations revealed a stable mixed chimerism, with the donor hematopoietic system co-existing with that of the recipient. Since the age of 13 years SBNE suffered from relapsing squamous cell cancers in the head and neck and gynaecological tract, either synchronous or metachronous carcinomas, requiring multiple surgical interventions. At the age of 19 years SBNE developed a locally advanced tumor of the oral cavity with regional metastases at presentation (compare MRT scans in Fig. 3). The optimal management of head and neck cancer in the setting of FA is not well established, but due to the increased risk of toxicity with chemotherapy, especially DNA cross-linking agents including cisplatin (the most commonly used and active agent in head and neck cancer), FA patients with head and neck cancer rarely receive chemotherapy. To estimate the extent of the DSB repair deficiency we analyzed her blood lymphocytes by the DNA damage foci approach. Since a significant proportion of the peripheral blood cells obviously originate from the patient (and not from the donor transplant), we were able to verify the substantial DSB repair defect, with about ∼6 excess foci at 8 h and ∼3 excess foci at 24 h post-irradiation compared to healthy control-children (Fig. 2B). In the light of this substantial DSB repair defect and her reduced general state of health (body weight of 14.3 kg at a height of 110 cm, recurrent syncopes due to hypoglycemia, renal and hepatic insufficiency) the decision was made for surgical intervention alone, without adjuvant radio- or chemotherapy. SBNE underwent subtotal glossectomy with bilateral neck dissection. Histopathological analysis revealed a moderately differentiated squamous cell carcinoma.

**Figure 4:**
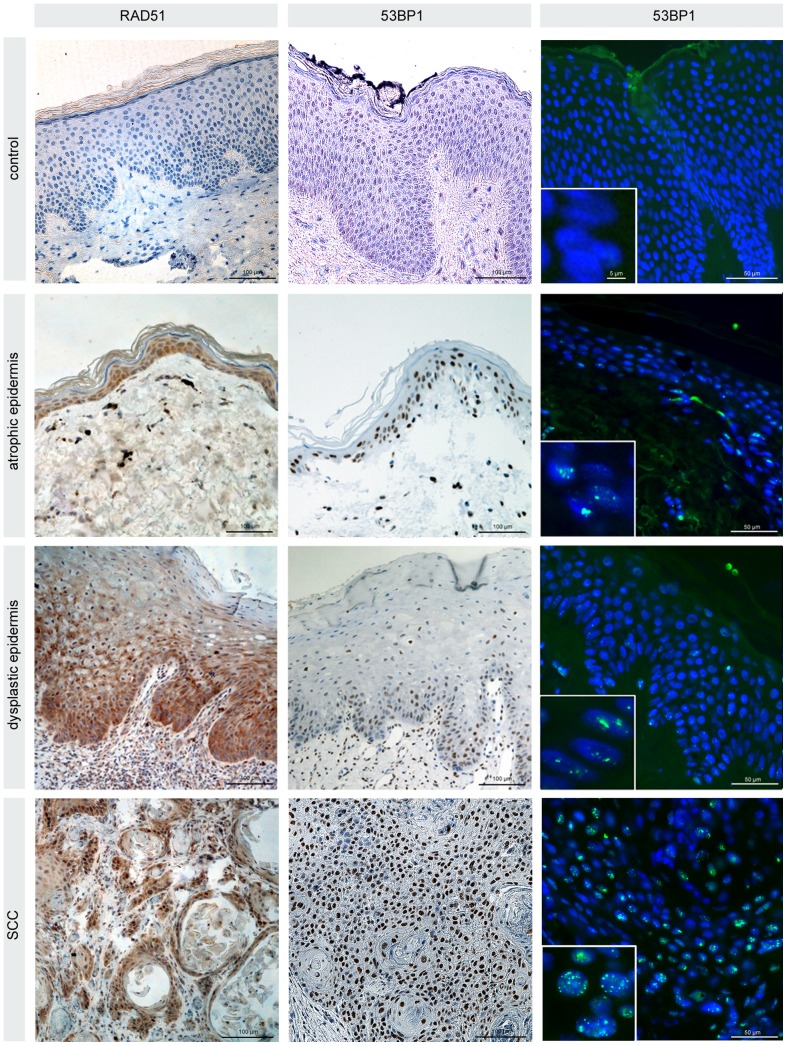
Light microscopy analysis of the skin and tumor obtained from the Fanconi patient. Immunohistochemical staining of RAD51 and 53BP1 (diaminobenzidine, brown) in atrophic and dysplastic epidermis as well as tumor specimens derived from the Fanconi patient. Compared with healthy control epidermis moderately or considerably increased RAD51 and 53BP1 expression in the proliferating zone attached to the basement membrane. Immunfluorescence staining of 53BP1 (green) reveals discrete nuclear foci in the proliferating cells of the epidermis, with increasing foci levels during tumor development. Original magnification, x600.

**Figure 5:**
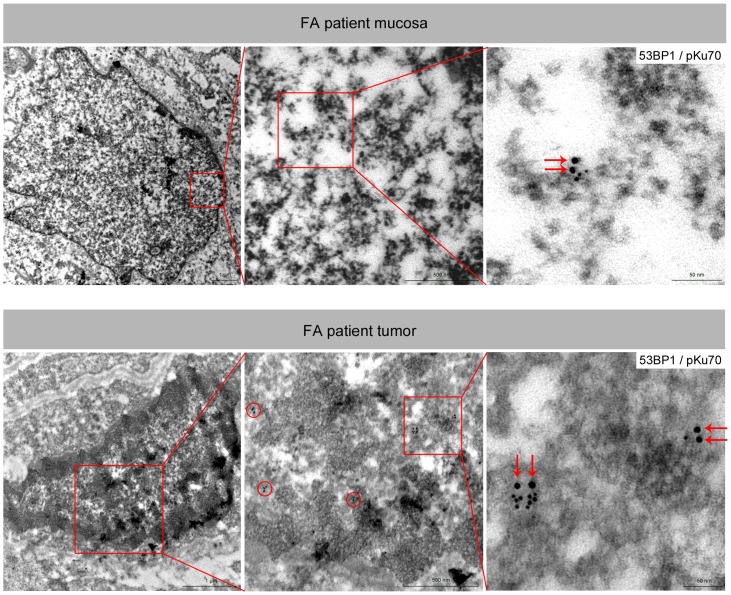
Electron microscopy analysis of the mucosa and tumor obtained from the Fanconi patient. TEM micrographs of double-labeling of pKu70 (10-nm beads) and p53BP1 (6-nm beads) at different magnifications (boxed regions are shown at higher magnifications in the following images). Co-localization of pKu70 and 53BP1 in electron-dense regions, reflecting actively processed DSBs in heterochromatin.

To gain insight into the mechanism of tumorigenesis we analyzed not only these tumor biopsies, but also specimens of the mucosa and skin for different DNA damage markers (Fig. 4). Although the precise mechanisms are unclear, cells lacking FA proteins are deficient in promoting HR activities. Recent studies revealed that key HR factors such as RAD51 and BRCA2 play important functions during DNA replication by protecting nascent DNA from degradation at stalled replication forks. Staining patient-derived skin specimens for RAD51, we observed highly increased RAD51 levels in the rapidly cycling cells of the epidermis (in the basal layers attached to the basement membrane), compared to specimens of healthy individuals (Fig. 4). These findings are consistent with data demonstrating RAD51 recruitment to stalled replication forks before interstrand crosslink processing, and emphasize the functional connection between FA and HR proteins at perturbed replication. Implying that NHEJ is the major pathway for removal of direct/immediate DSBs throughout the cell cycle we also analysed DNA damage marker associated with NHEJ. 53BP1 protects DNA ends from excessive resection in G1, and thereby favors repair by NHEJ as opposed to HR. Immunhistochemical and immunfluorescence staining revealed a marked increase of 53BP1 expression in the proliferating zone of the skin and mucosa (Fig. 4). To gain more detailed insight into the biological significance of 53BP1 foci accumulation, we established the immunogold-labeling of 53BP1 with pKu70 in combination with TEM. In previous studies this TEM approach has allowed the detection of phosphorylated Ku70 (pKu70) bound at radiation-induced DNA breaks. Pairs of gold beads separated by a distance of ∼15 nm, reflecting the two individual Ku70 molecules of the Ku heterodimer, have been shown to mark unrepaired DSBs [10], [11]. In contrast to tissue samples of healthy individuals, we observed a predominant co-localization of 53BP1 with pKu70 in the FA patient-derived mucosa and skin samples, suggesting an accumulation of actively processed DSBs exclusively in the heterochromatic compartments (Fig. 5).

Taken together, our findings of a defective DNA repair in non-proliferating blood lymphocytes after irradiation as well as DNA damage accumulation in rapidly cycling cells of normal tissues are in line with the conception that FA proteins play an essential role in chromatin remodeling, independent of the repair pathway choice. In the face of genotoxic stress and during DNA replication, chromatin has to be dismantled in the vicinity of DNA damage or ahead of the progressing replicative fork, and then after religation of the break or behind the fork faithfully reassembled in order to re-establish the correct chromatin structure. Analysing multiple tissue samples derived from this FA patient, the accumulation of unrepaired DSBs in proliferating cells of the mucosa and epidermis can be recognized as a causative factor for tumor development. The DNA damage accumulation appears to promote tumorigenesis through a multistep process with a sequence of various histopathologic stages (atrophic and dysplastic phases) and thus account for the high tumor susceptibility in FA patients.

## Discussion

Major advances in pediatric cancer therapy have resulted in substantial improvements in survival, but normal-tissue toxicities associated with complex multimodality treatment strategies may compromise the clinical outcome of affected children. In this clinical study, we show that the DNA damage foci analysis of irradiated blood lymphocytes allows to identify patients with an impaired DSB repair capacity as the determining factor for high-grade normal tissue toxicities. In a FA patient suffering relapsing squamous cell carcinomas of the oral cavity we also characterized the defective repair in biopsies of skin, mucosa and tumor by analyzing DNA damage response proteins by light and electron microscopy. We observed an excessive accumulation of heterochromatin-associated DSBs in rapidly cycling cells of the proliferative zone in skin and mucosa. The degree of DNA damage accumulation correlated with the different histopathological stages of tumor development, whereas the maximum of unrepaired DNA damage was observed in the tumor cells themselves.

In our former study patient-derived lymphocytes were tested directly after blood collection and lymphocyte isolation withdrawal [24]. In our present study, by contrast, patient-derived blood lymphocytes were stored in liquid nitrogen prior to in-vitro irradiation and subsequent foci analysis to implement this screening tool for a multicentric setting. Cryopreserved blood lymphocytes were evaluated whether the long-term storage at a temperature below −130°C may compromise the quality of the subsequent foci analysis. Moreover, we applied different DNA damage markers to analyze DSB repair and to compare the sensitivity and reliability of these different markers to detect DSB repair deficiencies. Testing cryopreserved lymphocytes of ATM^−/−^ homozygote and ATM^+/−^ heterozygote probands we were able to verify the profound DSB repair defect of ATM^−/−^ homozygotes, independent from the DNA damage marker used. However, the subtle, genetically determined DSB repair deficiency of ATM^+/−^ heterozygote probands was confirmed by γH2AX only at 8 h post-irradiation, but not by 53BP1 or pATM foci analysis. These findings suggest that the predictive capacity of the foci analysis may be hindered by the cryopreservation of blood lymphocytes and, beyond that, may depend on the specific DNA damage marker used. Since the slight impairment of ATM^+/−^ heterozygotes was identified more clearly in our former studies using whole blood samples or purified lymphocytes without cryopreservation, our results suggest that the cryopreservation of blood lymphocytes does not impede the verification of profound DSB repair defects, but may hinder the detection of subtle DSB repair impairments.

In our patient collective we observed 4 children (GRJN, HFLA, MRME, SHAA) with unexpected severe normal-tissue toxicities (grade 4), but only GRJN suffering eventually a lethal leukencephalopathy after combined radio-/chemotherapy revealed an impaired DSB repair capacity. The underlying molecular defect of this DSB repair impairment could not be identified. The foci analysis of the other children with grade-4 toxicities (HFLA, MRME, SHAA) was without pathological findings, suggesting that other pathomechanisms than defective DSB repair account for the formation of severe normal tissue toxicities.

Little is known about the etiology of childhood cancers, but it is now well accepted that genetic alterations in proteins participating in the DNA-damage response play a significant role in determining the individual's cancer susceptibility. In this study, we observed statistically significant differences in the mean DSB repair capacity between tumor-children and healthy control-children, even if the tumor-children with striking DSB repair deficiencies (GRJN, SBNE) were disregarded from statistical analysis. This finding suggests that childhood cancer in general might have a genetic basis affecting the DNA-damage response. Alterations in DSB repair genes may predispose these children to increased risks for cancer formation and may also render them more sensitive to DNA-damaging treatments [26].

FA is caused by mutations in a genetically and biochemically complex set of proteins, ensuring genomic integrity by controlling chromatin processing during the DNA damage response. The FA pathway is activated during DNA replication and by DNA damage in the form of interstrand cross-links and other DNA lesions. FA is largely characterized by cellular hypersensitivity to DNA damaging agents that induce DNA interstrand crosslinking, impairing strand separation and unwinding, and ultimately hindering replication and transcription.

Although the most common malignancies are hematologic, FA is also associated with an increased predisposition to non-hematologic tumors, particularly squamous cell carcinomas (SSC) of the aerodigestive and anogenital tract [27]. The reason for the strong propensity for FA patients to develop SSCs is unclear [28]. Analyzing tissue samples of a Fanconi patient suffering relapsing SSCs in the head and neck region, the immunohistochemical localization of RAD51 and 53BP1 in the proliferating zone of the epidermis and mucosa suggest that DNA damage may arise from replication stress, marking repair intermediates during the repair process [29]. Using our TEM approach to characterize these 53BP1 foci, we show that 53BP1 co-localize with pKu70 in the proliferating keratinocytes exclusively in heterochromatic regions, suggesting the formation replication-associated DSBs within complex heterochromatin. Chromatin remodelling is now being recognized as an essential component of DNA repair [10], [11], [15], [16], [17]. Eukaryotic genomic DNA is packed into a highly condensed chromatin structure where the repeating nucleosomes form the basic unit. Chromatin can be roughly divided into heterochromatin (silenced) or euchromatin (active) states that are defined by their degree of compaction as well as their occupancy by specific combinations of modified histones, which are involved in regulating chromatin formation. During DNA replication, chromatin has to be dismantled ahead of the progressing replicative fork, and then faithfully reassembled in heterochromatic or euchromatic form behind the fork in order to preserve genomic and epigenetic information [30]. Our current data provide novel insights regarding the molecular understanding of the FA disorder, suggesting a defective repair of replication-associated breaks. However, we still have a limited mechanistic understanding of how FA proteins facilitate DNA replication and damage response to promote genomic integrity, and suppress tumorigenesis [21].

Taken together, analysing patient-derived tissue samples our results show that deficiencies in the FA pathway are associated with irreparable DSBs in proliferating precursor cells leading to genomic instability, and appreciably increase the likelihood of tumor formation in mucosa and skin. Moreover, our data suggest that DSB repair alteration in children predispose to cancer formation and affect children's susceptibility to DNA-damaging radio- and chemotherapy. The DNA damage foci approach may serve as a predictive assay in the screening of children with malignant tumors to identify individuals with DNA repair deficiencies, and thus, are genetically predisposed to develop severe normal-tissue toxicities. However, not all treatment-associated high-grade toxicities can be explained by DNA repair deficiencies.
